# Smurf2-induced degradation of SMAD2 causes inhibition of hair follicle stem cell differentiation

**DOI:** 10.1038/s41420-022-00920-x

**Published:** 2022-04-04

**Authors:** Bojie Lin, Dan Huang, Guanyu Lin, Yong Miao, Jin Wang, Zhexiang Fan, Zhiqi Hu

**Affiliations:** 1 Department of Plastic and Aesthetic Surgery, Guangxi Key Laboratory of Enhanced Recovery after Surgery for Gastrointestinal Cancer, The First Affiliated Hospital of Guangxi Medical University, Nanning, 530021 PR China; 2 Department of Plastic and Aesthetic Surgery, Nanfang Hospital of Southern Medical University, Guangzhou, 510515 PR China

**Keywords:** Diseases, Cell biology

## Abstract

Hair follicle stem cells (HFSCs) are implicated in the formation of hair follicles and epidermis. This study aims to clarify the role of SMAD2 in regulating the differentiation of HFSCs, which is involved with Smurf2. Functional assays were carried out in human HFSCs to assess the effect of SMAD2 and Smurf2 with altered expression on growth dynamics of HFSCs. Ubiquitination of SMAD2 and its protein stability were assessed. The binding relationship between NANOG and DNMT1 was assessed. A mouse skin wound model was induced to verify the effects of Smurf2/SMAD2/NANOG/DNMT1 on wound healing. SMAD2 overexpression was observed in HFSCs during differentiation and its ectopic expression contributed to promotion of differentiation and apoptosis of HFSCs while arresting cell proliferation. Mechanistic investigations indicated that Smurf2 promoted the ubiquitination and degradation of SMAD2, thus causing downregulation of SMAD2 expression. By this mechanism, NANOG expression was reduced and the subsequent DNMT1 transcriptional expression was also diminished, leading to suppression of differentiation and apoptosis of HFSCs while stimulating cell proliferation. Moreover, in vivo data showed that Smurf2 upregulation limited epidermal wound healing in mice by inhibiting the SMAD2/NANOG/DNMT1 axis. Our work proposed a potential target regarding SMAD2 restoration in promoting HFSC differentiation and skin wound healing.

## Introduction

Human hair follicle stem cells (HFSCs) possess the important property of adult stem cells, such that they are multipotent and easily accessible [1, 2]. HFSCs can also differentiate into various cell types, including sweat gland cells, neurons, and epithelial stem cells, and consequently hold huge potential in regard to tissue repair [3–5]. More importantly, a recent study demonstrated that HFSCs may differentiate to epidermis phenotype and migrate into the wound site [6]. Therefore, it would be plausible to hypothesize that HFSCs could potentially advance skin wound healing in diseases such as diabetic ulcers and injuries in war. Moreover, adult bulge HFSCs are also known to contribute to hastening skin wound healing in rat models [7]. Furthermore, hair follicle-fated bulge stem cells can augment transient wound re-epithelialization [8].

Mothers against decapentaplegic homolog 2 (SMAD2) is well-known for its role in regulation in differentiation of numerous cell types. For instance, SMAD2 functions as a promoter of TGF-β differentiation of neural crest cells into vascular smooth muscle cells and pulp cells [9, 10]. SMAD2 has also been reported to promote the differentiation of HFSCs into SMCs by serving as a transcription regulator of TGF-β [11]. In addition, a previous study reported that SMAD2 could induce the activation of NANOG to maintain the primed pluripotency of stem cells [12]. NANOG, a homeobox protein, itself serves as a transcriptional factor in the maintenance of pluripotency of stem cells. Interestingly, NANOG has also been demonstrated to promote the differentiation of HFSCs into SMCs, which is similar as SMAD2 [13]. Furthermore, NANOG has been shown to directly-bind to the promoter of DNA methyltransferase 1 (DNMT1) and upregulate DNMT1 [14]. Therefore, DNMT1 could serve as a downstream signaling molecule participating in NANOG-mediated HFSC differentiation. DNMT1, a key modulator of DNA methylation, mediates cell differentiation in multiple cell types, including bone mesenchymal stromal cells and hematopoietic stem cells [15, 16]. In light of these results, we speculated that SMAD2 might function in HFSC differentiation and skin wound healing via NANOG-mediated expression of DNMT1.

In addition to downstream signaling molecules, SMAD2 has also been previously shown to be degraded by SMAD-specific E3 ubiquitin protein ligase 2 (Smurf2) through the process of ubiquitination [17]. TGF-β-mediated epithelial-mesenchymal transition and myofibroblast differentiation are crucial steps in normal tissue repair, while Smurf2 can downregulate TGF-β signaling proteins including SMAD2/3 through ubiquitination and degradation [18]. This poses the question that whether SMAD2-mediated HFSC differentiation could be regulated by Smurf2. As a result, the current study set out to investigate the role of the Smurf2/SMAD2/NANOG/DNMT1 axis in wound healing using mouse skin wound models.

## Results

### SMAD2 promotes the differentiation and apoptosis of HFSCs while inhibiting their proliferation

**Fig. 1:**
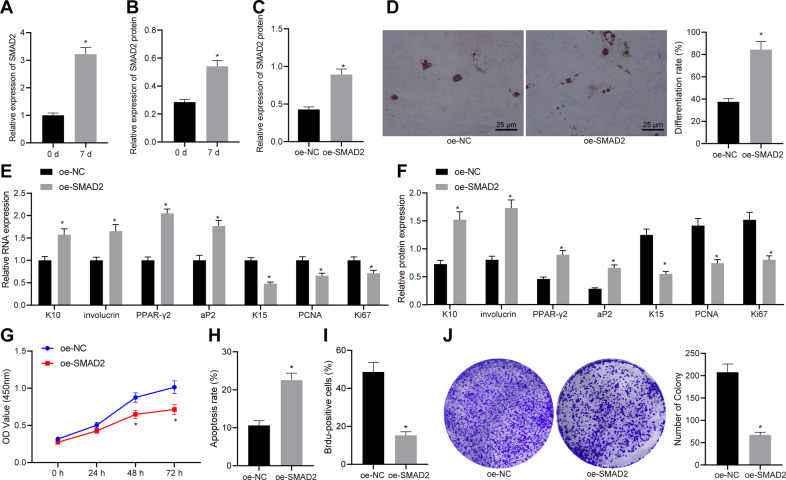
SMAD2 is upregulated in HFSCs after differentiation and its overexpression promotes the differentiation of HFSCs but suppresses cell proliferation. SMAD2 mRNA (**A**) and protein (**B**) levels in HFSCs after 7-day induction of cell differentiation measured by RT-qPCR and western blot assay, with β-actin as internal reference, *p < 0.05 vs. day 0. **C** SMAD2 protein level in HFSCs transduced with lentivirus-packaged oe-SMAD2. **D** Cell differentiation determined by oil red O staining (×400). mRNA (**E**) and protein (**F**) levels of epidermal differentiation markers (K10 and involucrin), adipogenesis markers (PPAR-γ2 and aP2), keratinocyte-specific marker (K15), and proliferation-related markers (PCNA and Ki67) in HFSCs overexpressing SMAD2 measured by RT-qPCR and western blot assay, with β-actin as internal reference. **G** Cell viability in HFSCs in response to SMAD2 overexpression determined by CCK-8 assay. **H** Apoptosis of HFSCs in response to SMAD2 overexpression determined by flow cytometry. **I** Proliferation of HFSCs in response to SMAD2 overexpression assessed by BrdU labeling. **J** The number of colonies formed in HFSCs in response to SMAD2 overexpression. *p < 0.05 vs. oe-NC group. Data are expressed as mean ± standard deviation. Data comparison between two groups was performed by unpaired *t* test, while data between multiple groups were compared by one-way ANOVA with Tukey’s post hoc test. Data at different time points were compared by two-way ANOVA with Bonferroni post hoc test. Each cell experiment was repeated 3 times.

Identification results demonstrated that the HFSCs isolated from hair follicles were scattered in hair follicles in the shape of stripes (200 μm) and that CD44, CD73, CD90, and CD105 were positive for HFSCs, with CD31 being negative (Supplementary Fig. 1A–C), which indicated successful isolation of HFSCs. Subsequently, by conducting RT-qPCR and western blot assay, the mRNA (Fig. 1A) and protein (Fig. 1B) levels of SMAD2 in the HFSCs were found to be notably higher on day 7 of differentiation induction relative to day 0. After HFSCs were transduced with lentivirus expressing SMAD2 for 48 h and screened by puromycin, the total protein content was extracted. As expected, infection with lentivirus-packaged oe-SMAD2 was noted to augment the expression of SMAD2 (Fig. 1C). SMAD2 overexpression also increased the differentiation rate of HFSCs (Fig. 1D). Consequently, SMAD2 overexpression increased the mRNA and protein levels of epidermal differentiation markers (K10 and involucrin) and adipogenesis markers (PPAR-γ2 and aP2), while decreasing those of keratinocyte-specific marker K15 and proliferation-related markers (PCNA and Ki67) (Fig. 1E, F), suggesting enhanced differentiation but reduced proliferation of HFSCs. Moreover, it was found that SMAD2 overexpression also reduced the proliferation of HFSCs, but enhanced their apoptosis (Fig. 1G–J). The above results indicated that SMAD2 overexpression promoted the differentiation of HFSCs but suppressed cell proliferation.

### Smurf2 degrades SMAD2 protein through ubiquitination

**Fig. 2:**
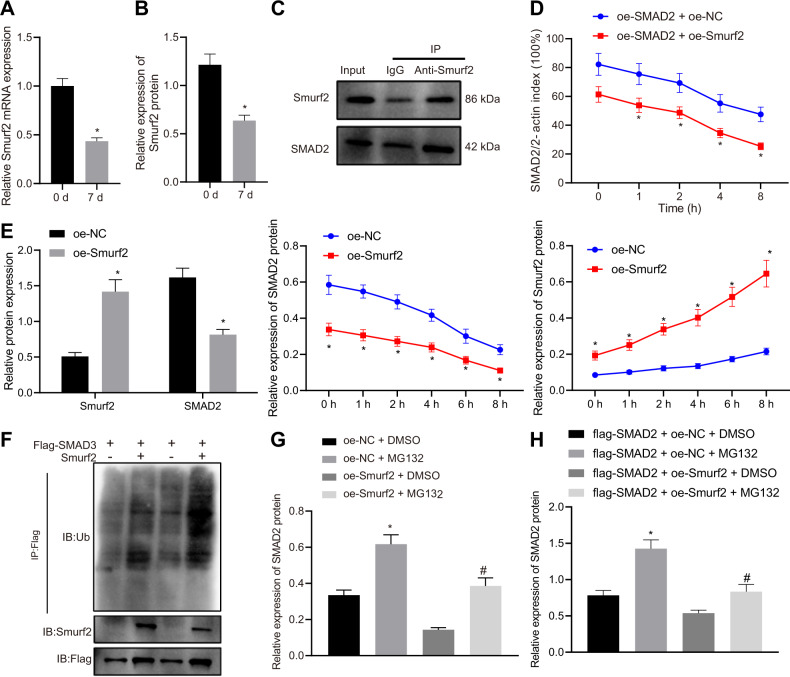
Smurf2 degrades SMAD2 through ubiquitination and then downregulates its expression. Smurf2 mRNA (**A**) and protein (**B**) levels in HFSCs after 7-day differentiation determined by RT-qPCR and western blot assay, with β-actin as internal reference, *p < 0.05 vs. day 0. **C** Interaction between Smurf2 and SMAD2 in HFSCs identified by IP. **D** Stability of SMAD2 protein in response to Smurf2 overexpression in HEK293 cells treated with cycloheximide for 1, 2, 4, 6, 8 h. *p < 0.05 vs. oe-SMAD2 + oe-NC group. **E** SMAD2 protein level in HFSCs overexpressing Smurf2 measured by western blot assay, with β-actin as internal reference, *p < 0.05 vs. oe-NC grou*p*. **F** Ubiquitination of SMAD2 in HFSCs after Smurf2 overexpression determined by IP (IB-Ub refers to ubiquitinated antibody used in immunoblot, while IB-SMAD2 refers to SMAD2 antibody used in immunoblot). **G** The ubiquitination of SMAD2 in HFSCs treated with proteasome inhibitor MG132 or DMSO alone or in the presence of Smurf2 determined by IP. *p < 0.05 vs. oe-NC + DMSO group; ^#^*p* < 0.05 vs. oe-Smurf2 + DMSO group. **H** The ubiquitination of SMAD2 in HFSCs in response to MG132 treatment and SMAD2 overexpression or in the presence of Smurf2 determined by IP. *p < 0.05 vs. flag-SMAD2 + oe-NC + DMSO group; ^#^*p* < 0.05 vs. flag-SMAD2 + oe-Smurf2 + DMSO group. Data are expressed as mean ± standard deviation. Data comparison between two groups was performed by unpaired *t* test, while data between multiple groups were compared by one-way ANOVA wi*t*h Tukey’s post hoc test. Data at different time points were compared by two-way ANOVA with Bonferroni post hoc test. Each cell experiment was repeated 3 times.

As a specific E3 ubiquitin ligase, Smurf2 is known to degrade the TGF-β signaling protein SMAD2 through the process of ubiquitination, and consequently reduce its transcriptional activity [17]; hence, we speculated whether SMAD2 could be mediated by Smurf2 during the differentiation of HFSCs. It was found that Smurf2 mRNA (Fig. 2A) and protein (Fig. 2B) levels were markedly reduced after 7 days of HFSC differentiation. In addition, SMAD2 protein was noted to be enriched in the complex pulled-down by anti-Smurf2 antibody, as revealed by immunoprecipitation (IP) assay, suggesting that Smurf2 may interact with the SMAD2 protein (Fig. 2C). Subsequently, SMAD2 protein levels were found to be reduced in the presence of overexpression of both SMAD2 and Smurf2 in HEK293 cells (Fig. 2D). Similarly, in HFSCs, Smurf2 overexpression was observed to reduce the protein levels of SMAD2 (Fig. 2E). As previously reported, Smurf2 can enhance the ubiquitination level of SMAD3 [17]. Hence, SMAD3 was utilized as a positive control, and an IP assay was performed to determine the ubiquitination levels of SMAD2 in the presence of Smurf2. The obtained findings demonstrated that Smurf2 overexpression induced the ubiquitination of SMAD2 and thus reduced its protein levels (Fig. 2F).

To further verify Smurf2-mediated SMAD2 ubiquitination, MG132 (a proteasome inhibitor) was introduced into the cells overexpressing Smurf2 alone or cells overexpressing both Smurf2 and SMAD2. As expected, the treatment with MG132 augmented the SMAD2 protein levels, which indicated that MG132 treatment diminished the Smurf2-induced ubiquitination and degradation of endogenous SMAD2 (Fig. 2G). Moreover, in the cells overexpressing both Smurf2 and SMAD2, MG132 treatment was found to further elevate the protein levels of SMAD2 (Fig. 2H), suggesting that MG132 inhibited the Smurf2-mediated ubiquitination and degradation of exogenous SMAD2. Together, these findings indicated that Smurf2 could degrade SMAD2 through ubiquitination and then downregulate its expression.

### Smurf2 degrades SMAD2 to inhibit the HFSC differentiation and induce cell proliferation

**Fig. 3:**
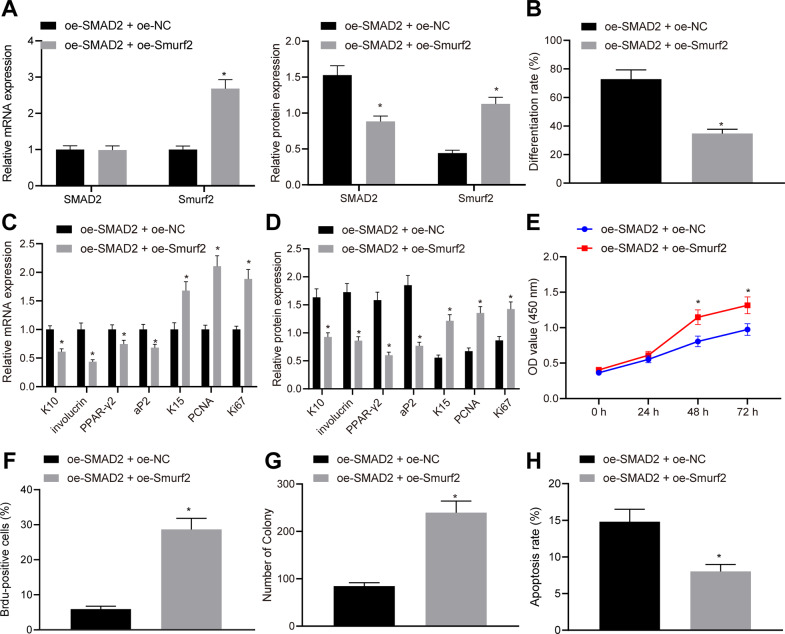
Smurf2 degrades SMAD2 to inhibit the differentiation of HFSCs and promote their proliferation. **A** SMAD2 and Smurf2 mRNA and protein levels in HFSCs overexpressing SMAD2 alone or transduced with lentivirus-packaged oe-Smurf2 in combination determined by RT-qPCR and western blot assay, with β-actin as internal reference. **B** Cell differentiation determined by oil red O staining. mRNA (**C**) and protein (**D**) levels of epidermal differentiation markers (K10 and involucrin), adipogenesis markers (PPAR-γ2 and aP2), keratinocyte-specific marker (K15), and proliferation-related markers (PCNA and Ki67) in HFSCs after SMAD2 overexpression alone or Smurf2 overexpression in combination measured by RT-qPCR and western blot assay, with β-actin as internal reference. **E** Cell viability after SMAD2 overexpression alone or Smurf2 overexpression in combination determined by CCK-8 assay. **F** Cell proliferation in response to SMAD2 overexpression alone or Smurf2 overexpression in combination assessed by BrdU labeling. **G** The number of colonies formed in HFSCs after SMAD2 overexpression alone or Smurf2 overexpression in combination. **H** Cell apoptosis in response to SMAD2 overexpression alone or Smurf2 overexpression in combination determined by flow cytometry. *p < 0.05 vs. oe-SMAD2 + oe-NC group. Data are expressed as mean ± standard deviation. Data comparison between two groups was performed by unpaired *t* test, while data between multiple groups were compared by one-way ANOVA with Tukey’s post hoc test. Data at different time points were compared by repeated measures ANOVA with Bonferroni post hoc test. Each cell experiment was repeated 3 times.

The effect of Smurf2-mdeiated degradation of SMAD2 through ubiquitination on the function of HFSCs was the next focus of this study. The results of RT-qPCR and western blot assay showed no obvious changes in the SMAD2 mRNA expression, reduced SMAD2 protein levels and increased mRNA and protein levels of Smurf2 in cells treated with oe-SMAD2 + oe-Smurf2 (Fig. 3A). In addition, cell differentiation was decreased in HFSCs overexpressing Smurf2 relative to cells overexpressing SMAD2 alone (Fig. 3B). Also, Smurf2 overexpression reduced the mRNA (Fig. 3C) and protein (Fig. 3D) levels of epidermal differentiation markers K10 and involucrin, and adipogenesis markers PPAR-γ2 and aP2, while increasing those of keratinocyte-specific markers K15 and proliferation-related markers PCNA and Ki67 in HFSCs overexpressing SMAD2. The results further demonstrated that Smurf2 overexpression enhanced the viability (Fig. 3E), proliferation (Fig. 3F) and colony formation abilities (Fig. 3G) of HFSCs overexpressing SMAD2, accompanied with suppressed apoptosis (Fig. 3H). These results suggested that Smurf2 impeded the apoptosis and differentiation of HFSCs while stimulating cell proliferation through degradation of SMAD2.

### SMAD2 upregulates NANOG to elevate DNMT1 transcriptional expression

**Fig. 4:**
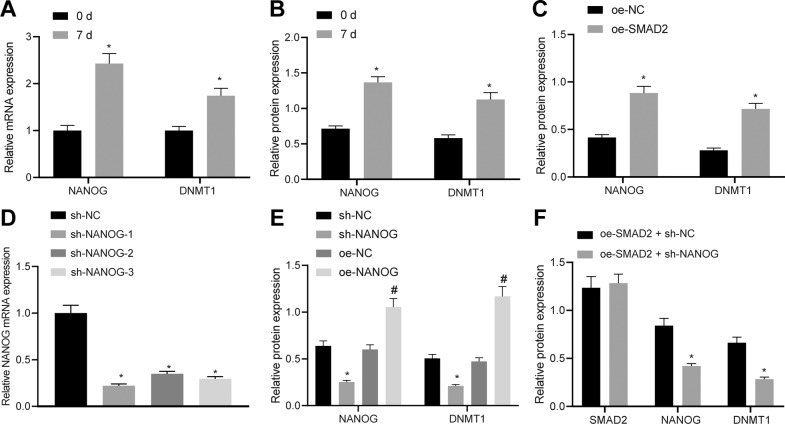
SMAD2 upregulates NANOG to increase the transcriptional expression of DNMT1. NANOG and DNMT1 mRNA (**A**) and protein (**B**) levels in HFSCs after 7-day differentiation determined by RT-qPCR and western blot assay, with β-actin as internal reference, *p < 0.05 vs. day 0. **C** NANOG and DNMT1 protein levels in HFSCs after SMAD2 overexpression measured by western blot assay, with β-actin as internal reference, *p < 0.05 vs. oe-NC group. **D** Silencing efficiency of 3 siRNAs targeting NANOG in HFSCs determined by RT-qPCR, with β-actin as internal reference, *p < 0.05 vs. sh-NC grou*p*. **E** NANOG and DNMT1 protein levels in HFSCs after overexpression or silencing of NANOG measured by western blot assay, with β-actin as internal reference, *p < 0.05 vs. sh-NC group; ^#^*p* < 0.05 vs. oe-NC group. **F** SMAD2, NANOG and DNMT1 protein levels in HFSCs overexpressing SMAD2 determined by western blot assay, with β-actin as internal reference, *p < 0.05 vs. oe-SMAD2 + sh-NC group. Data are expressed as mean ± standard deviation. Data between multiple groups were compared by one-way ANOVA with Tukey’s post hoc test. Each cell experiment was repeated 3 times.

SMAD2 possesses the ability to induce the activation of NANOG [12], which can stimulate the differentiation of HFSCs into SMCs [13]. As previously reported, NANOG can directly bind to the promoter of DNMT1 and upregulate its expression [14]. As a result, we shifted our focus to investigating whether NANOG and DNMT1 were implicated in the differentiation of HFSCs. It was found that NANOG and DNMT1 mRNA (Fig. 4A) and protein (Fig. 4B) levels were significantly elevated in HFSCs after 7 days of HFSC differentiation. In addition, overexpression of SMAD2 augmented the protein levels of NANOG and DNMT1 (Fig. 4C).

Subsequently, three siRNAs were selected to knock-down NANOG in HFSCs. The results of RT-qPCR displayed that all siRNAs, sh-NANOG-1, sh-NANOG-2 and sh-NANOG-3 brought about notable reductions in NANOG mRNA levels in HFSCs (Fig. 4D), wherein sh-NANOG-1 exhibited the highest silencing efficiency and therefore, was selected for subsequent experiments. It was found that NANOG silencing reduced the DNMT1 protein levels in HFSCs, whereas overexpression of NANOG increased DNMT1 protein levels (Fig. 4E). Furthermore, dual-luciferase reporter assay was performed to identify their relationship and the results of which showed that NANOG overexpression enhanced the luciferase activity of DNMT1-wildtype (WT) without affecting that of DNMT1-mutant (MUT) (Supplementary Fig. 2A), suggesting that NANOG could enhance the expression of DNMT1 by binding to DNMT1 promoter. Meanwhile, the results of chromatin immunoprecipitation (ChIP) assay demonstrated that the enrichment of the DNMT1 promoter immunoprecipitated by NANOG antibody was markedly increased when compared to the IgG antibody (Supplementary Fig. 2B), indicating that NANOG bound to the promoter of DNMT1 and promoted its transcription. To further verify this finding, NANOG was silenced in the HFSCs overexpressing SMAD2, and the western blot assay results showed that silencing NANOG reduced the DNMT1 protein levels in the presence of SMAD2 (Fig. 4F). These data suggested that SMAD2 increased the transcriptional expression of DNMT1 by upregulating the expression of NANOG.

### SMAD2 promotes HFSC differentiation and apoptosis while inhibiting cell proliferation by upregulating NANOG and DNMT1

**Fig. 5:**
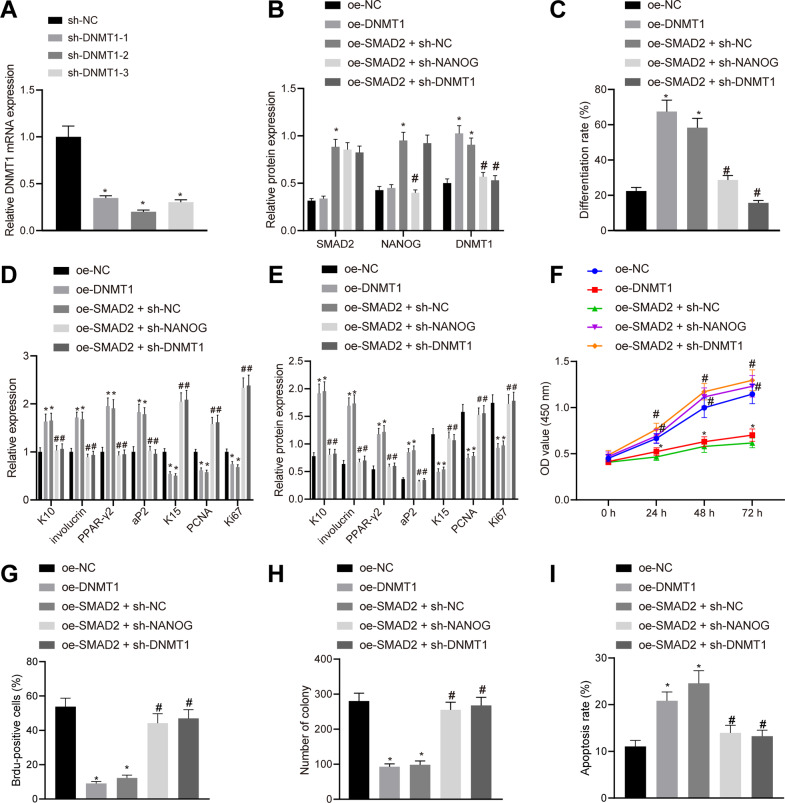
SMAD2 promotes HFSC differentiation and apoptosis while suppressing cell proliferation by activating the NANOG/DNMT1 axis. **A** Silencing efficiency of 3 siRNAs targeting DNMT1 in HFSCs determined by RT-qPCR, with β-actin as internal reference. *p < 0.05 vs. sh-NC group. **B** SMAD2, NANOG, and DNMT1 protein levels in HFSCs after different treatments measured by western blot assay, with β-actin as internal reference. **C** Differentiation rate of HFSCs after different treatments determined by oil Red O staining. mRNA (**D**) and protein (**E**) levels of epidermal differentiation markers (K10 and involucrin) and adipogenesis markers (PPAR-γ2 and aP2), keratinocyte-specific marker (K15), and proliferation-related markers (PCNA and Ki67) in HFSCs after different treatments determined by RT-qPCR and western blot assay, with β-actin as internal reference. **F** Cell viability after different treatments determined by CCK-8 assay. **G** Cell proliferation after different treatments assessed by BrdU labeling. **H** The number of colonies formed in HFSCs after different treatments. **I** Cell apoptosis after different treatments determined by flow cytometry, *p < 0.05 vs. oe-NC group; ^#^*p* < 0.05 vs. oe-SMAD2 + sh-NC group. Data are expressed as mean ± standard deviation. Data between multiple groups were compared by one-way ANOVA with Tukey’s post hoc test while data at different time points were compared by two-way ANOVA with Bonferroni post hoc test. Each cell experiment was repeated 3 times.

Additionally, three siRNAs were constructed to silence DNMT1 in HFSCs. All three siRNAs, sh-DNMT1-1, sh-DNMT1-2, and sh-DNMT1-3 brought about reductions in DNMT1 mRNA levels (Fig. 5A), wherein sh-DNMT1-2 (sh-DNMT1) exhibited the highest silencing efficiency and therefore, was selected for subsequent experiments.

We then over-expressed DNMT1 or SMAD2 alone in the HFSCs, or silenced DNMT1 or NANOG in the HFSCs overexpressing SMAD2 to analyze their regulation in the functions of HFSCs. It was found that the protein levels of SMAD2, NANOG, and DNMT1 were all elevated in HFSCs transduced with lentivirus-packaged oe-SMAD2. Meanwhile, sh-DNMT1, as expected, decreased the DNMT1 protein levels in HFSCs overexpressing SMAD2, while oe-DNMT1 brought about the opposite findings. Also, NANOG silencing was found to down-regulate the DNMT1 protein levels in HFSCs overexpressing SMAD2 (Fig. 5B). In addition, SMAD2 overexpression or DNMT1 overexpression increased the differentiation of HFSCs. On the other hand, reduced cell differentiation was noted in HFSCs overexpressing SMAD2 when either NANOG or DNMT1 were silenced (Fig. 5C). Moreover, SMAD2 overexpression or DNMT1 overexpression increased the mRNA and protein levels of epidermal differentiation markers and adipogenesis markers, while decreasing those of keratinocyte-specific marker and proliferation-related markers. Whereas, reduced mRNA and protein levels of epidermal differentiation markers and adipogenesis markers but elevated levels of keratinocyte-specific marker and proliferation-related markers were observed in HFSCs overexpressing SMAD2 when either NANOG or DNMT1 were silenced (Fig. 5D, E). Furthermore, SMAD2 overexpression or DNMT1 overexpression reduced the viability at 48th and 72nd h (Fig. 5F), proliferation (Fig. 5G) and colony formation abilities (Fig. 5H) of HFSCs, and restored their apoptosis to initial level (Fig. 5I). Taken together, SMAD2 could induce the differentiation and apoptosis of HFSCs and inhibit their proliferation by activating the NANOG/DNMT1 axis.

### Smurf2 inhibits epidermal wound healing in mice via inactivation of the SMAD2/NANOG/DNMT1 axis

**Fig. 6:**
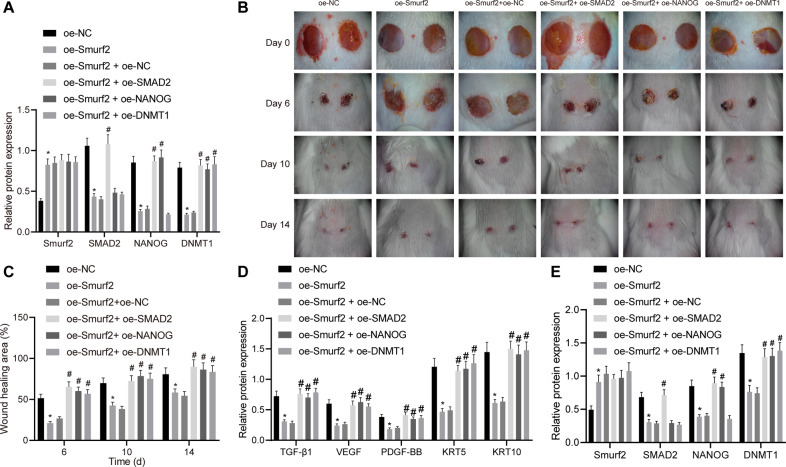
Smurf2 degrades SMAD2 to prevent the activation of NANOG and DNMT1 and hence to reduce epidermal wound healing in mice. **A** Smurf2, SMAD2, NANOG and DNMT1 protein levels in the wounded tissues from mice on day 10 measured by western blot assay. **B** Representative images of wound area in mice on the days 0, 6, 10, and 14. **C** Wound healing area in mice on the days 0, 6, 10, and 14. **D** Protein expression of growth factors TGF-β1, VEGF, PDGF-BB, KRT5 and KRT10 in the wounded tissues from mice measured by western blot assay. **E** Smurf2, SMAD2, NANOG and DNMT1 protein levels in the wounded tissues from mice on day 18 measured by western blot assay. *p < 0.05 vs. oe-NC group; ^#^*p* < 0.05 vs. oe-Smurf2 + oe-NC group. Data are expressed as mean ± standard deviation. Data between multiple groups were compared by one-way ANOVA with Tukey’s post hoc test. *n* = 6.

Lastly, mouse models of wound were developed to test the aforementioned findings in vivo. Western blot assay results revealed that Smurf2 overexpression brought about reductions in the protein levels of SMAD2, NANOG and DNMT1 in the skin tissues from mouse wound model, while SMAD2 overexpression reversed the reductions in NANOG and DNMT1 levels caused by Smurf2 overexpression, and NANOG overexpression rescued the protein levels of DNMT1 inhibited by Smurf2 (Fig. 6A).

Additionally, different extents of epidermal wound healing were observed in mice. It was found that Smurf2 overexpression decreased the wound healing area on days 6, 10, and 14. Meanwhile, either SMAD2 or NANOG overexpression could reverse the inhibitory effect of Smurf2 overexpression on the wound healing area (Fig. 6B, C). On day 18 after wounding, the skin tissues at the wounded site were extracted, and the data obtained from western blot assay demonstrated that Smurf2 overexpression decreased the levels of growth factors TGF-β1, VEGF, PDGF-BB, KRT5 and KRT10 in the wounded tissues, whereas SMAD2 or NANOG overexpression countered the effect of Smurf2 on these factors (Fig. 6D). In addition, western blot assay results presented that oe-Smurf2 treatment elevated the protein levels of Smurf2 while reducing those of SMAD2, NANOG and DNMT1 in the skin tissues from mouse wound model, while further treatment with oe-SMAD2 reversed these effects. Treatment with oe-Smurf2 + oe-NANOG caused higher protein levels of NANOG and DNMT1 than treatment with oe-Smurf2 + oe-NC. DNMT1 protein levels were noted to be elevated in the presence of oe-Smurf2 + oe-DNMT1 relative to oe-Smurf2 + oe-NC (Fig. 6E). Altogether, these findings suggest that Smurf2 downregulates the SMAD2/NANOG/DNMT1, thereby reducing mouse epidermal wound healing.

## Discussion

**Fig. 7:**
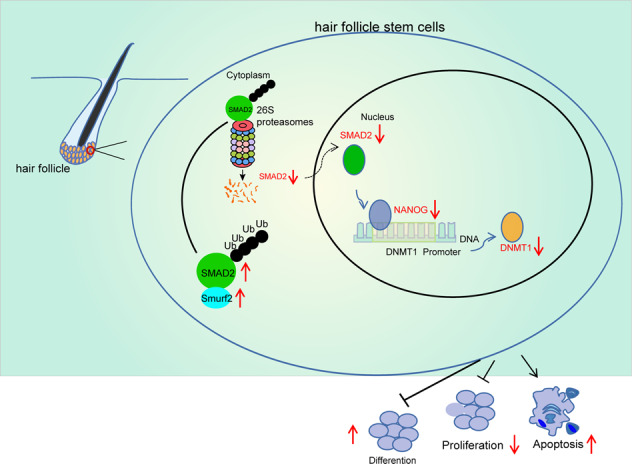
A schematic map showing the involvement of Smurf2/SMAD2/NANOG/DNMT1 axis in the HFSC differentiation, proliferation and apoptosis. Smurf2 degrades SMAD2 through ubiquitination, resulting in prevention of NANOG expression and DNMT1 transcriptional expression, thereby suppressing HFSC differentiation and apoptosis but inducing cell proliferation, ultimately inhibiting epidermal wound healing.

Our data suggested some important findings. First of all, our findings demonstrated SMAD2 was highly-expressed during differentiation of HFSCs, wherein overexpression of SMAD2 enhanced the differentiation of HFSCs, while reducing their proliferation and colony formation abilities. Secondly, we found that Smurf2, as the E3 ubiquitin protein ligase, Smurf2 brought about the degradation of SMAD2 and therefore, reduced HFSC differentiation. Thirdly, further experimentation in our study unveiled that SMAD2 bound to NANOG and upregulated NANOG expression levels, which bound to DNMT1 and augmented its expression, consequently enhancing cell differentiation of HFSCs (Fig. 7). Altogether, these findings suggest that SMAD2 enhanced the differentiation of HFSCs through upregulation of NANOG and DNMT1. Moreover, Smurf2 overexpression reduced SMAD2 to prevent the activation of NANOG and DNMT1 in mouse skin and to restrain wound healing in vivo.

Early large HFSCs are essential for the regeneration of the interfollicular epidermis and also function in early skin morphogenesis, with some studies even suggesting the use of HFSCs are promising cell sources for wound healing [19, 20]. More recently, HFSCs have been elaborated to play critical roles in the process of faster re-epithelialization and partial-thickness burn wound healing [21]. Expanding on the current understanding of HFSCs, we demonstrated that involvement of the SMAD2/NANOG/DNMT1 axis in HFSC differentiation, and further highlighted the promotive role of SMAD2 in HFSC differentiation. SMAD2 is known to serve as an important transcription regulator of the TGF-β signaling pathway, responsible for inducing the differentiation of hair follicle mesenchymal stem cells (MSCs) [22]. In addition, HFSCs that are unable to respond to TGF-β signals can precipitate the delayed regeneration of hair follicles [23]. More importantly, studies have shown that SMAD2 can promote TGF-β-mediated differentiation of HFSCs, which is in line with our findings [11]. Collectively, results obtained in our study indicate that SMAD2 possess pro-differentiation activity, at least in HFSCs.

Another instrumental finding in the current study was that SMAD2 augmented the expression of NANOG, which is in agreement with a previous study that suggested SMAD2 can bind to regulatory promoter sequences to activate NANOG [12]. Inherently, NANOG serves as a transcription factor that maintains the pluripotency of stem cells [24, 25]. Shedding more light on their functions, we found that NANOG promoted the differentiation of HFSCs. This particular finding is in line with a previous study that demonstrated magnetofection-mediated NANOG delivery can enhance the differentiation of hair follicle MSCs [13]. More notably, Activin A treatment and upregulation of SMAD2/3 can also elevate the level of porcine NANOG [26]. On the other hand, downregulation of NANOG has also been associated with the inhibitory effect of TNF-α on the differentiation of MSCs to sweat glands [27]. Further in line with our findings, one particular study demonstrated that NANOG directly-binds to the promoter of DNMT1 and upregulates DNMT1, thereby enhancing the differentiation potential of MSCs [14]. Additionally, we uncovered that DNMT1 promoted the differentiation of HFSCs, further highlighting DNMT1 as a downstream signaling molecule of SMAD2-mediated differentiation. DNMT1 has been shown to promote differentiation in multiple cell types such as bone mesenchymal stromal cells [15], hematopoietic stem cells [16], and results from our study provide evidence for one more cell type, HFSCs.

Furthermore, we found that Smurf2 degraded SMAD2 through ubiquitination, a finding that has also been documented earlier [17]. This result suggests that SMAD2-mediated differentiation of HFSC and skin wound healing could be suppressed by Smurf2. Smurf2, a SMAD-specific ubiquitin E3 ligase, controls TGF-β signaling proteins such as the TGF-β receptor and SMAD2/3 [28]. Smurf2 can also bring about degradative polyubiquitylation of SMAD1 [29]. Similarly, SMAD2 and SMAD7 are renoprotective in the course of renal fibrosis, wherein Smurf2 stimulates the degradation of SMAD2, resulting in weakened or repressed biological function [30]. More recent studies suggest that tetratricopeptide repeat domain 3 promotes myofibroblast differentiation through the inhibition of Smurf2-dependent suppression of SMAD2/3 [18]. Lastly, our in vivo experimentation findings confirmed that Smurf2 gain-of-function reduced SMAD2 to restrain HFSC differentiation into epidermis and repress wound healing.

Nevertheless, there is a notable limitation in the current study. We found that the SMAD2/NANOG/DNMT1 axis was involved in HFSC differentiation and skin wound healing in separate experiments. Although our results suggested that HFSCs may differentiate into epidermis for wound healing, further studies should be performed to confirm that HFSCs can enhance skin wound healing. Besides, whether this axis affects other functions of HFSCs such as adhesion during wound healing also remains to be explored in future endeavors.

Altogether, findings obtained in the current study indicate that SMAD2 can promote the differentiation of HFSCs through upregulation of NANOG and DNMT1. These signaling molecules may be of therapeutic value in facilitating wound healing in diseases such as diabetic ulcer. Also, we found that SMAD2 can be degraded by Smurf2, while Smurf2 overexpression inhibited wound healing in vivo. It is therefore possible that Smurf2 deficiency may be a therapeutic target for enhanced wound healing, such as in diabetic ulcer.

## Materials and methods

### Ethics statement

The collection of scalp tissue samples was approved by the Clinical Ethics Committee of First Affiliated Hospital of Guangxi Medical University and human experiments were conducted following the *Declaration of Helsinki*. Informed consent was obtained from each individual. The animal use and experimental procedures were approved by the Animal Ethics Committee of First Affiliated Hospital of Guangxi Medical University.

### Isolation and identification of HFSCs

Scalp tissue specimens were obtained from the 18 patients with scalp lacerations and bruises (10 males, 8 females) at First Affiliated Hospital of Guangxi Medical University. The obtained scalp tissues were washed 3 times with penicillin-containing Hank’s solution, followed by 2 washes with penicillin-free Hank’s solution. Next, the tissues were sliced into 2 × 2 mm sections, and digested with 0.48 U/ml of neutral protease overnight at 4 °C. Complete hair follicle was collected using surgical forceps under a microscope. Subsequently, the hair follicles were digested with mixture of 0.05% trypsin and 0.02% EDTA solution on a 37 °C shaker for 30 min. The detachment was terminated with the addition of fetal bovine serum (FBS). The mixture was filtered through a 100-mesh steel mesh and then centrifuged at 1000 rpm for 5 min. The supernatant was discarded and the cell pellets were cultured with DMEM:F12-3:1 culture medium supplemented with insulin (5 mg/L), transferrin (5 mg/L), hydrocortisone (0.4 mg/L), EGF (10 ng/mL), amphotericin B (2.5 mg/L), penicillin (1051 U/L), streptomycin (100 mg/L), and 20% FBS (C0265, Beyotime, Shanghai, China). After culture for 48 h at 70% confluence, the cells were counted and seeded in a T25 flask at a density of 2 × 10^5^ cells/mL, cultured and passaged at 37 °C, 5% CO_2_ in saturated humidity. The identification of HFSCs was performed using immunofluorescence staining [31].

### Differentiation of HFSCs

Adipogenic differentiation assay of HFSCs was conducted as previously described [32]. Briefly, HFSCs were cultured in adipogenic differentiation medium of DMEM/F12 (Life Technologies) containing 10% FBS (Hyclone), 1 mM dexamethasone, 0.5 mM isobutylmethylxanthine, 10 mM insulin, and 200 mM indomethacin (Sigma-Aldrich, MO, USA). After 14 days of culture, cell differentiation was identified by detecting the expression patterns of epidermal differentiation markers (K10 and involucrin) and adipogenic markers (PPAR-γ2, aP2), keratinocyte-specific marker K15 and proliferation markers (PCNA and Ki67) using RT-qPCR and western blot assay.

### Cell transduction

Upon reaching 30% confluence, the cells were transduced with lentivirus-packaged SMAD2 overexpression plasmid (oe-SMAD2), Smurf2 overexpression plasmid (oe-Smurf2), NANOG overexpression plasmid (oe-NANOG), DNMT1 overexpression plasmid (oe-DNMT1), siRNAs targeting NANOG (sh-NANOG-1, sh-NANOG-2, sh-NANOG-3), siRNA targeting DNMT1 (sh-DNMT1) and corresponding negative control (oe-NC, sh-NC) (Genechem, Shanghai, China). Corresponding lentivirus at 2 × 10^6^ TU (MOI: 1), 5 μg polybrene, 1 mL of serum-free DMEM/F12 medium were then mixed for cell transduction. Cell transduction efficiency was subsequently observed by percentage of cells carrying fluorescence under an inverted fluorescence microscope for 2–3 days. After 48 h of transduction, 1 μg/mL of puromycin was added to each well to select the stably transduced cells, which were finally cultured in conventional medium [33]. Stably transduced cell lines were established using lentivirus infection. The lentivirus was packaged using 293T cells.

### Oil red O staining

Keratinocytes can produce adipose tissue. Oil red O staining of lipid droplets can reflect the degree of differentiation of HFSCs into keratinocytes [34]. HFSCs were cultured in DMEM/F12 medium at 37 °C for 12 h and then cultured in DMEM/F12 medium containing 20% FBS. Next, the cells were differentiated on the surface of 1% agar for 14 days, fixed with 10% formalin, 60% isopropanol, and stained with oil red O solution. After staining, the cells were rinsed with 60% isopropyl alcohol and fixed with glycerin gelatin. Subsequently, the number of oil red O positive cells was counted under a microscope (Olympus Optical Co., Ltd, Tokyo, Japan).

### CCK-8 assay

CCK-8 assay was employed to assess the viability of HFSCs. Specifically, HFSCs that underwent 48 h of transduction were detached and resuspended after screening by puromycin. Cell concentration was adjusted to 1 × 10^5^ cells/mL, seeded into a 96-well plate at 100 μL/well, and then cultured overnight. The cells were subsequently treated according to the instructions of the CCK-8 assay kit (Beyotime, Shanghai, China). CCK-8 detection solution (10 μL) was added for 4-h incubation. Absorbance at 450 nm was measured using a microplate reader. Cell viability was detected at 24, 48, and 72 h after seeding and a growth curve was plotted.

### Flow cytometry

Flow cytometry was adopted to assess cell apoptosis in HFSCs. Briefly, HFSCs that underwent 48 h of transduction were detached and resuspended after screening by puromycin. Cell suspension (1 × 10^5^ cells/mL) was cultured in a 96-well plate at 100 μL/well overnight. The subsequent procedures were performed in accordance with the instructions of APOPTEST^TM^-fluorescein isothiocyanate (FITC), wherein the cells were rinsed with PBS and resuspended in binding buffer, and then stained with Annexin-V FITC and propidium iodide (PI) for 10 min. Lastly, cell apoptosis was detected using a BD FACSCanto flow cytometer (Becton-Dickinson, San Jose, CA).

The HFSC surface markers were detected by flow cytometry. The cells were incubated with primary antibodies (Abcam, Cambridge, MA, USA) against CD90 (ab226123, 1:50), CD31 (ab222783, 1:100), CD44 (ab189524, 1:1000, Abcam), CD73 (ab133582, 1:100), and CD105 (ab221675, 1:500), and then with the fluorescent secondary antibody goat anti-rabbit IgG H&L (Alexa Fluor^®^ 488) (ab150077, 1:500, Abcam), followed by detection using the FACSCanto flow cytometer (BD Biosciences, San Jose, CA, USA). Repeated and unstained samples were used as a NC. FACSDiva Version 6.1.3 (BD Biosciences) and FlowJo 10.1 software (Tree Star, Ashland, OR, USA) were used to analyze data.

### BrdU labeling

Cells were seeded in a 35 mm petri dish with a cover glass at a density of 1.5 × 10^5^ cells/mL, cultured for 1 day, and synchronized with 0.4% FCS medium for 3 days, so that most of the cells were at the G0 phase. Before terminating the cell culture, BrdU (final concentration of 30 μg/L) was added to the cells for incubation at 37 °C for 40 min. The culture medium was discarded, and the cover glass was washed 3 times with PBS. Cells were fixed with methanol/acetic acid for 10 min, air-dried and treated with 0.3% H_2_O_2_-methanol for 30 min to inactivate the endogenous oxidase. Next, the cells were blocked with 5% normal rabbit serum and reacted with primary antibody, namely anti-mouse BrdU monoclonal antibody (1:50), and NC (with PBS or serum added) and detected using the ABC method. The cells were stained with hematoxylin or eosin, and observed under a microscope where the total number of cells and the number of BrdU-positive cells in 10 high-power fields were randomly counted. The positive rate of BrdU was calculated.

### Colony formation assay

The colony formation ability was assessed in vitro using a soft agar colony formation assay. Briefly, cells at the logarithmic phase of growth were dispersed into a single-cell suspension. Each 60 mm petri dish was then seeded with 1000 cells and cultured in a 5% CO_2_ incubator. Culture medium was changed every 3 days. After 14 days of culture, the medium was discarded. Subsequently, the cells were rinsed 3 times with PBS, fixed with methanol for 15 min, and stained with crystal violet for 15 min. Finally, the number of the colonies (more than 50 cells in each colony) was counted under a microscope.

### Western blot assay

The tissues or cells were collected, and lysed in PMSF-containing lysis buffer (RIPA, protease inhibitor, and phosphatase inhibitor = 100: 1: 1) on ice for 30 min. Next, the cell lysates were centrifuged at 10,000 rpm at 4 °C for 15 min, and the supernatant was collected and transferred to new micro-tube. Protein concentration was then determined using a BCA kit (Thermo Fisher Scientific, Waltham, MA). Protein (30 μg) was subjected to polyacrylamide gel electrophoresis at a constant 80 V for 35 min and 120 V for 45 min. After electrophoresis, the proteins were transferred to a PVDF membrane (Amersham, Piscataway, NJ) and blocked with 5% skim milk at room temperature for 1 h. After removing the blocking solution, the membranes were incubated with primary antibodies against Smurf2 (rabbit monoclonal antibody, dilution ratio of 1:1000, ab94483, Abcam, Cambridge, UK), SMAD2 (rabbit monoclonal antibody, dilution ratio of 1:2000, ab40855, Abcam), NANOG (rabbit monoclonal antibody, dilution ratio of 1:1000, ab109250, Abcam), DNMT1 (rabbit polyclonal antibody, dilution ratio of 1:1000, ab87654, Abcam), TGF-β1 (rabbit polyclonal antibody, dilution ratio of 1:200, ab92486, Abcam), vascular endothelial growth factor (VEGF) (rabbit polyclonal antibody, dilution ratio of 1:200, ab2350, Abcam), platelet-derived growth factor (PDGF)-BB (rabbit polyclonal antibody, dilution ratio of 1:200, ab9704, Abcam), keratin 15 (KRT15, rabbit monoclonal antibody, dilution ratio of 1:10,000, ab52816, Abcam), keratin 10 (KRT10, rabbit monoclonal antibody, dilution ratio of 1:10,000, ab76318, Abcam), Flag (M2) (Sigma-Aldrich), involucrin (dilution ratio of 1:10,000, ab181980, Abcam), PPAR-γ2 (dilution ratio of 1:1000, ab41928, Abcam), aP2 (dilution ratio of 1: 25,000, ab76007, Abcam) and β-actin (rabbit polyclonal antibody, dilution ratio of 1:2000, ab8227, Abcam) at 4 °C overnight. The membranes were washed 3 times with PBST (PBS buffer containing 0.1% Tween-20) for 10 min each. Afterwards, the horseradish peroxidase-labeled secondary goat anti-rabbit IgG (H&L, dilution ratio of 1:2000, ab6721, Abcam) was added for 1-h incubation at room temperature. After incubation, the membranes were washed 3 times with PBST buffer for 10 min each. Following development with an optical luminometer (GE, Boston, MA), grayscale of each protein band was measured using the Image Pro Plus 6.0 software (Media Cybernetics, Silver Spring, MD) with β-actin serving as the loading control.

### RT-qPCR

TRIzol (16096020, Thermo Fisher Scientific) was applied to extract total RNA content from tissues or cells. cDNA (5 µg) was then obtained in accordance with the instructions of cDNA synthesis kit (K1622; Fermentas Inc., Ontario, CA). Using the cDNA as a template, real time qPCR was performed according to the instructions of TaqMan Gene Expression Assay protocol (Applied Biosystems, Foster City, CA). All samples were tested in triplicates. Primer sequences are shown in Supplementary Table 1. Relative mRNA expression normalized to β-actin was calculated using the 2^−ΔΔCT^ method.

### IP assay

IP assay was performed for detecting SMAD2 ubiquitination level. In brief, HFSCs were lysed with lysis buffer (P0013, Beyotime) on ice for 30 min. The cell lysate was collected into a 1.5 mL microtube and centrifuged at 12,000 *g* at 4 °C for 15 min. Protein A and protein G beads (50 µL each) were then mixed in a 1.5 mL micro-tube. Protein A + G agarose beads (10 µL) and Smurf antibody (ab94483, Abcam) were incubated with the cell lysate overnight at 4 °C. After IP reaction, the mixture was centrifuged at 3000 rpm at 4 °C for 3 min. Agarose beads were carefully rinsed with 1 mL of lysis buffer. The immunoprecipitants were subsequently boiled with 2× SDS loading buffer (15 µL) for 5 min. Changes in SMAD2 ubiquitination levels were then determined using western blot assay.

### ChIP assay

ChIP assay was performed to validate the enrichment of DNMT1 promoter. Cells were fixed with 4% paraformaldehyde and incubated with glycine for 10 min to produce DNA-protein crosslinking. Next, the cells were lysed with cell lysis buffer and nuclear lysis buffer (contained in ChIP kit; EMD Millipore) and sonicated to produce 200–300 bp chromatin fragments. The lysate was subsequently immunoprecipitated with magnetic protein A beads bound to the NANOG antibody (ab109250, Abcam). Rabbit anti-IgG antibody (ab171870, Abcam) was used as a negative control. The precipitated DNA was analyzed with RT-qPCR with primer sequence listed in Supplementary Table 2.

### Protein stability test

Human embryonic kidney cell line HEK293 (American Type Culture Collection, Manassas, VA, USA) were cultured in DMEM containing 10% FBS and 1% penicillin-streptomycin in a 5% CO_2_ incubator at 37 °C. NANOG and DNMT1 were over-expressed in HEK293 cells. After 36 h of transduction, the cells were treated with cycloheximide (50 μg/mL; CalBiochem, Gibbstown, NJ). The cells harvested at different time points (0, 1, 2, 4, 6, 8 h) were lysed with RIPA lysis buffer for protein content extraction. The expression patterns of DNMT1 protein at each time point were measured using western blot assay.

### Dual-luciferase reporter assay

In order to test the binding of NANOG to the DNMT1 promoter, DNMT1 promoter region which bound to NANOG (5'-TGGCAATTACCCCGT-3') and the mutation fragment with mutated NANOG binding site (5'-TGGCATACGTCCCGT-3') were cloned into the psiCheck2 vector. Also, oe-NC and oe-NANOG were co-transfected with recombinant luciferase reporter vector into HEK293 cells. After 48 h of incubation, the cells were lysed and assayed with Dual Luciferase Reporter Assay System (Promega) to measure Firefly luciferase activity, with Renilla luciferase activity as the internal reference.

### Mouse wound model

A total of 36 mice (weighing 20-24 g; Hunan SJA Laboratory Animal Co., Ltd., Hunan, China) were housed individually in the SPF laboratory at 22–25 °C and 60–65% humidity under a 12-h light/dark cycle, with ad libitum access to food and water. The mice were acclimated for 1 week before experiment. The health of the mice was observed before the experiment. The mice were anesthetized by intraperitoneal injection of 3% pentobarbital sodium. After removing the hair on the back, the skin was disinfected with iodine. After deiodination, two round wounds of 1.5 cm diameter were made 1.0 cm away both sides of the posterior edge of the mouse spine using a circular punch, avoiding touching the muscles. After the wound was formed, the mice were administered medication without wound dressing. The mice were individually housed in a sterile laboratory and sterilized daily. Subsequently, the lentivirus (5 × 10^8^ pfu/100 μL) was introduced into the site next to the wound via injection. The mice were assigned into oe-NC, oe-Smurf2, oe-Smurf2 + oe-NC, oe-Smurf2 + oe-SMAD2, oe-Smurf2 + oe-NANOG, and oe-Smurf2 + oe-DNMT1 groups (6 mice/group). Lentiviral vector LV5-GFP was utilized for gene overexpression, and lentiviral vector pSIH1-H1-copGFP was utilized for gene silencing. Mouse wounds were imaged and the wound area was recorded on days 6, 10 and 14. On the day 18 after modeling, the skin tissues were collected from the wound area and paraffin-embedded.

### Immunofluorescence staining

Differentiated HFSCs were fixed overnight with 4% paraformaldehyde, and rinsed with 0.01 M PBS three times. The cells were blocked with 10% goat serum at room temperature for 30 min, and then incubated with antibodies (Abcam) to CD90 (ab226123, 1: 100), CD31 (ab222783, 1: 100), CD44 (ab189524, 1: 4000), CD73 (ab133582, 1: 50), and CD105 (ab221675, 1: 1000) at 4 °C overnight. Next, the cells were re-probed with the secondary antibody goat anti-rabbit IgG H&L (Alexa Fluor^®^ 488) (ab150077, 1: 500, Abcam), stained with DAPI for 1 h under dark conditions at room temperature and fixed with glycerol. The images of co-localization in cells were captured using a confocal laser scanning microscope (FV1000; Olympus).

### Statistical analysis

Statistical analyses were performed using the SPSS 21.0 software (IBM Corp. Armonk, NY). Measurement data was expressed as mean ± standard deviation. Comparison of two sets of data was performed by unpaired *t* test. Data comparison among multiple groups was performed by one-way analysis of variance (ANOVA), followed by Tukey’s post-hoc test. Data comparison between groups at different time points was performed using two-way ANOVA with Bonferroni post-hoc test. A value of *p* < 0.05 was considered statistically significant.

## Supplementary information

Supplemental Materials

## Data Availability

The datasets generated/analysed during the current study are available.
