# Supplementary material for: Smurf2-induced degradation of SMAD2 causes inhibition of hair follicle stem cell differentiation
Source: Cell Death Discov. 2022 Apr 4;8:160. doi: 10.1038/s41420-022-00920-x (PMC8980066; doi:10.1038/s41420-022-00920-x)
Supplement: Supplementary file 1 — Supplemental Materials [file 41420_2022_920_MOESM1_ESM.docx]

**Supplemental Materials**

**Supplementary Fig. 1** Identification of HFSCs. A, The distribution of HFSCs in hair follicle under an inverted microscope (scale bar = 50 µm). B, The HFSC surface markers detected by immunofluorescence (scale bar = 25 µm). C, The HFSC surface markers detected by flow cytometry.

**
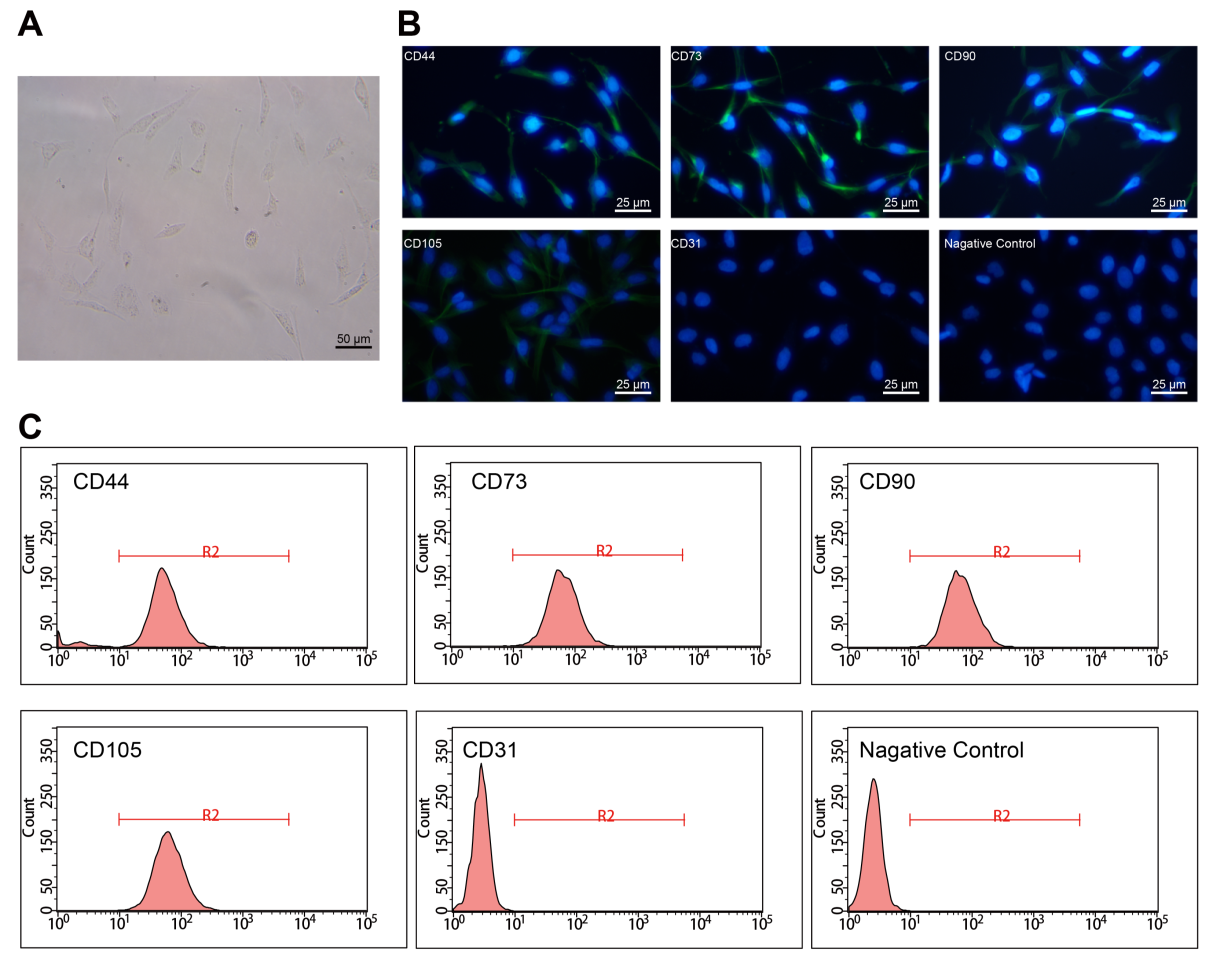
**

**Supplementary Fig. 2** Verification of the binding of NANOG to the DNMT1 promoter. A, Binding relationship between NANOG and DNMT1 in HEK293 cells identified by dual-luciferase reporter assay with DNMT1 promoter region which bound to NANOG and the mutation fragment with mutated NANOG binding site cloned into the psiCheck2 vector. **p* < 0.05 *vs*. oe-NC group. B, Relative enrichment of DNMT1 promoter immunoprecipitated by anti-NANOG antibody determined by ChIP assay, **p* < 0.05 *vs*. IgG group.

**
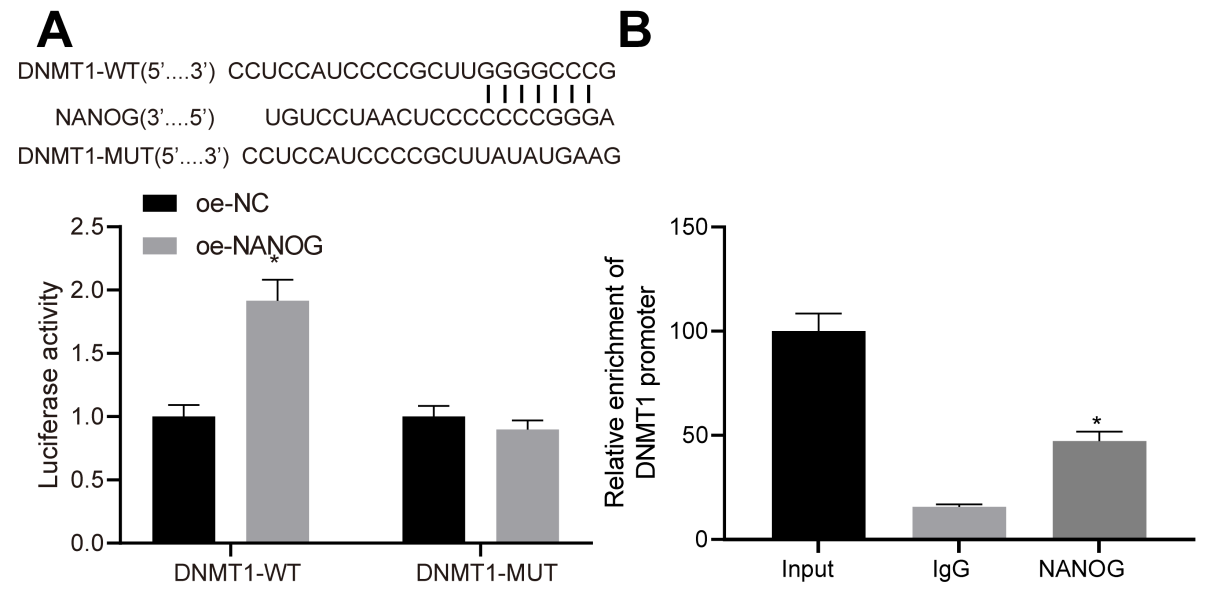
**

**Supplementary Table 1** Primer sequences for RT-qPCR

| Gene | Primer sequence |
| --- | --- |
| K10 | F: 5’-TTGCTGAACAAAACCGCAAAG-3’ |
|  | R: 5’-GCCAGTTGGGACTGTAGTTCT-3’ |
| Involucrin | F: 5’-GACTGCTGTAAAGGGACTGCC-3’ |
|  | R: 5’-CATTCCCAGTTGCTCATCTCTC-3’ |
| PPAR-γ2 | F: 5’-TGGAGCCCAAGTTTGAGTT-3’ |
|  | R: 5’-CAATCTGTCTGAGGTCTG-3’ |
| aP2 | F: 5’-CCACGCTTAACCTGGGTGG-3’ |
|  | R: 5’-AGGACTTGATGTTTGACTCC-3’ |
| perilipin2 | F: 5’-CACCGTGGCCATGTGGAT-3’ |
|  | R: 5’-CCCTCCGTGTCTGTCTGGTC-3’ |
| Adipoq | F: 5’-TGAGAAGGGTGAGAAAGGAG-3’ |
|  | R: 5’-TAGGCACCTTCTCCAGGTTC-3’ |
| K15 | F: 5’-AATGATCCAGACCAGCAAG-3’ |
|  | R: 5’-CAGTGAGTTCTCCAGCC-3’ |
| PCNA | F: 5’-GTAGTAAAGATGCCTTCTGGTG-3’ |
|  | R: 5’-TCTCTATGGTAACAGCTTCCTC-3’ |
| Ki67 | F: 5’-AAGACAGTGTTGCTCAGGGAA-3’ |
|  | R: 5’-AGTTGGGTCTCCCCCTGTAA-3’ |
| Smurf2 | F: 5’-GGGAGCGCCCAACACGAC-3’ |
|  | R: 5’-ATTACGGATCTCCCATCC-3’ |
| SMAD2 | F: 5’-TCACAGCCCTCACTCACTGTA-3’ |
|  | R: 5’-ATTTCTACCGTGGCATTTC-3’ |
| NANOG | F: 5’-GAGATGCCTCACACGGAGAC-3’ |
|  | R: 5’-GGTCTGGTTGCTCCACATTG-3’ |
| DNMT1 | F: 5’-ACCGCTTCTACTTCCTCGAGGCCTA-3’ |
|  | R: 5’-GTTGCAGTCCTCTGTGAACACTGTGG-3’ |
| β-actin | F: 5’-AGCTACGAGCTGCCTGACG-3’ |
|  | R: 5’-CGTGGATGCCACAGGACTC-3’ |

Note: RT-qPCR, reverse transcription quantitative polymerase chain reaction; F, forward, R, reverse.

**Supplementary Table 2** Primer sequences for ChIP-qPCR

| Primer sequence | Forward | Reverse |
| --- | --- | --- |
| DNMT1 promoter | 5’-CCCCCACACACTGGGTATAG-3’ | 5’-AGGGTTTGTGAGAGCCCTTG-3’ |

**Original western blots of Figure 2C**

**
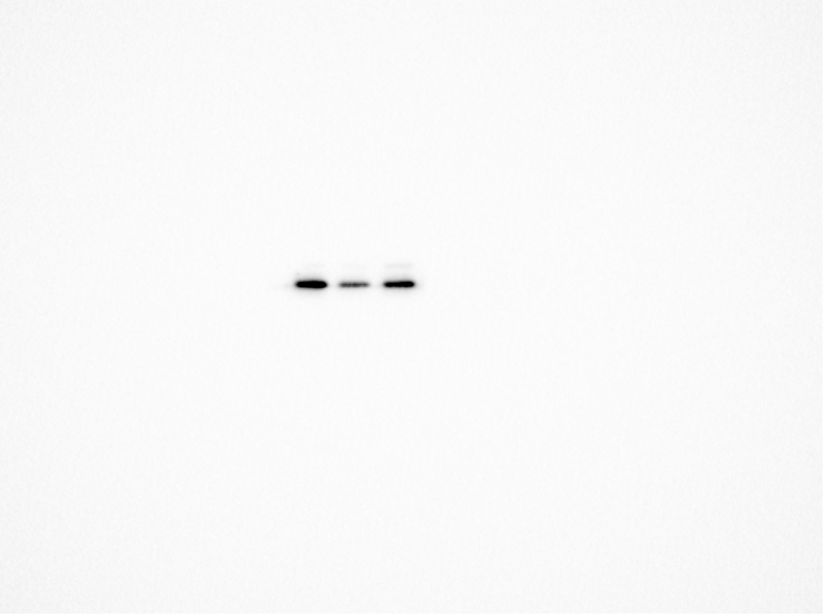
Smurf2**

**
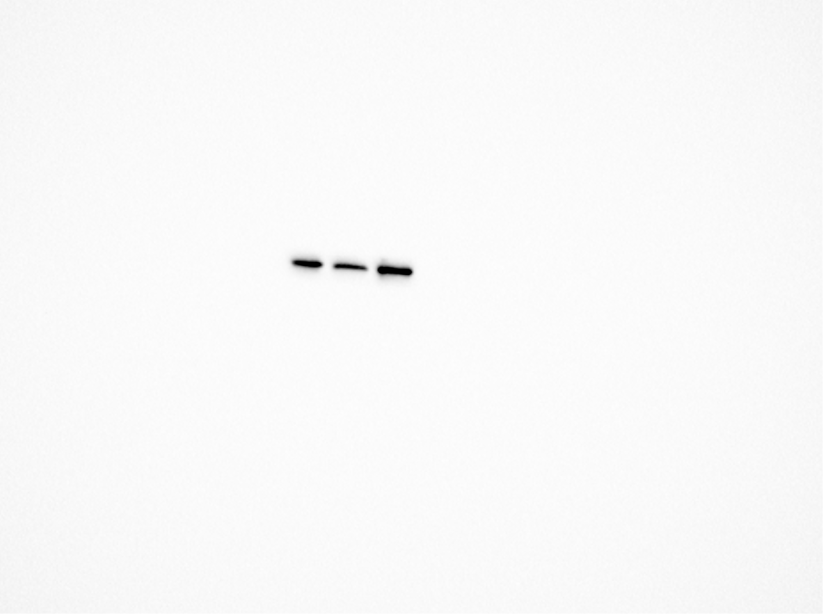
SMAD2**

**Original western blots of Figure 2F**

**
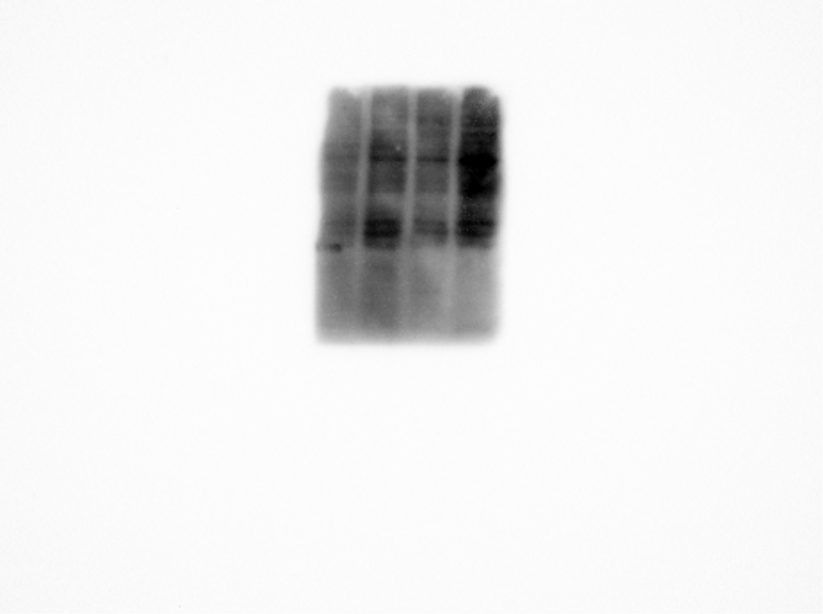
IB:Ub**

**
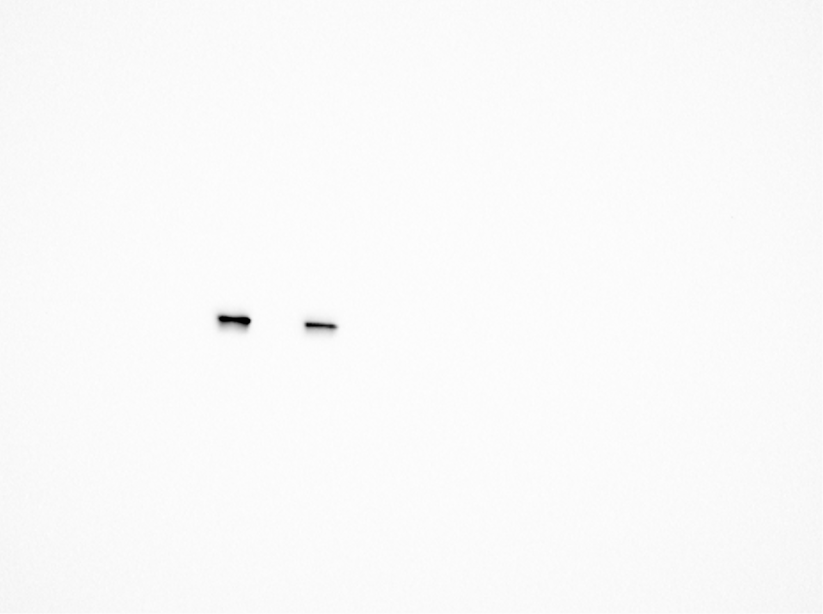
IB:Smurf2**

**
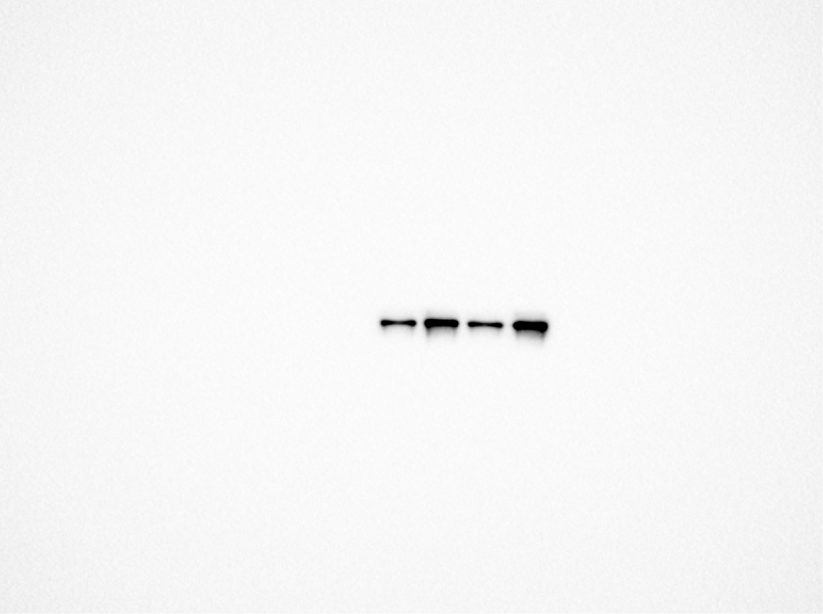
IB:Flag**
